# Salt stress response triggers activation of the jasmonate signaling pathway leading to inhibition of cell elongation in Arabidopsis primary root

**DOI:** 10.1093/jxb/erw202

**Published:** 2016-05-23

**Authors:** Camilo E. Valenzuela, Orlando Acevedo-Acevedo, Giovanna S. Miranda, Pablo Vergara-Barros, Loreto Holuigue, Carlos R. Figueroa, Pablo M. Figueroa

**Affiliations:** ^1^Instituto de Ciencias Biológicas, Universidad de Talca, Talca 3465548, Chile; ^2^Escuela de Biotecnología, Facultad de Ciencias, Universidad Santo Tomás, Santiago 8370003, Chile; ^3^Departamento de Genética Molecular y Microbiología, Facultad de Ciencias Biológicas, Pontificia Universidad Católica de Chile, Santiago 8331010, Chile

**Keywords:** Arabidopsis, jasmonate, osmotic stress, root, salt, signaling.

## Abstract

Salinity is a severe abiotic stress that affects irrigated croplands. Jasmonate (JA) is an essential hormone involved in plant defense against herbivory and in responses to abiotic stress. However, the relationship between the salt stress response and the JA pathway in *Arabidopsis thaliana* is not well understood at molecular and cellular levels. In this work we investigated the activation of JA signaling by NaCl and its effect on primary root growth. We found that JA-responsive *JAZ* genes were up-regulated by salt stress in a COI1-dependent manner in the roots. Using a JA-Ile sensor we demonstrated that activation of JA signaling by salt stress occurs in the meristematic zone and stele of the differentiation zone and that this activation was dependent on JAR1 and proteasome functions. Another finding is that the elongation zone (EZ) and its cortical cells were significantly longer in JA-related mutants (*AOS*, *COI1*, *JAZ3* and *MYC2/3/4* genes) compared with wild-type plants under salt stress, revealing the participation of the canonical JA signaling pathway. Noteworthy, osmotic stress – a component of salt stress – inhibited cell elongation in the EZ in a COI1-dependent manner. We propose that salt stress triggers activation of the JA signaling pathway followed by inhibition of cell elongation in the EZ. We have shown that salt-inhibited root growth partially involves the jasmonate signaling pathway in Arabidopsis.

## Highlight

This study identifies that salt-inhibited root growth partially involves the activation of the jasmonate signaling pathway in Arabidopsis.

## Introduction

Soil salinity, the build-up of salts at or near the surface of the soil, is a widespread agricultural problem affecting the world’s irrigated cropland (Yamaguchi-Shinozaki and Shinozaki, 2006). Salinity is a major abiotic stress factor that limits crop production due to ionic, osmotic and oxidative stresses with negative impact on plant growth (Munns and Tester, 2008). The negative effects of salt on plants come from an excess of sodium ions that has a detrimental effect on biochemical reactions and water availability triggering osmotic stress (Zhu, 2002).

Hormones play a fundamental role in the plant’s ability to adapt to environmental changes such as abscisic acid (ABA), a hormone known to be involved in abiotic stress response and tolerance (Peleg and Blumwald, 2011). Increasing evidence supports the idea that jasmonate (JA) can play relevant functions in abiotic stress response (Kazan, 2015; Riemann *et al.*, 2015). The JA-mediated responses are dependent on COI1, an F-box protein member of the SCF^COI1^ ubiquitin-ligase complex (Xie *et al.*, 1998). The SCF^COI1^ complex is involved in the ubiquitin-dependent degradation of *JA*SMONATE *Z*IM-DOMAIN (JAZ) proteins in the presence of the biologically active JA-Ile (Chini *et al.*, 2007; Thines *et al.*, 2007; Yan *et al.*, 2007; Fonseca *et al.*, 2009). JAZ proteins bind to bHLH transcription factors (e.g. MYC2, MYC3, MYC4 and MYC5) that are activators of JA responses repressing their transcriptional activity and turning off the expression of the early JA-responsive genes (Chini *et al.*, 2007; Cheng *et al.*, 2011; Fernández-Calvo *et al.*, 2011; Niu *et al.*, 2011; Figueroa and Browse, 2012, 2015; Qi *et al.*, 2015; Zhang *et al.*, 2015). JAR1 (jasmonate resistant 1) is a JA-amido synthetase that catalyzes conversion of JA to JA-Ile, which is recognized by the COI1-JAZ co-receptor (Staswick and Tiryaki, 2004; Sheard *et al.*, 2010). Immediately after destabilizing JAZ by action of JA-lle, transcription factors such as MYC2, MYC3 and MYC4 are released from repression activating early JA responses (Cheng *et al.*, 2011; Fernández-Calvo *et al.*, 2011; Niu *et al.*, 2011; Zhang *et al.*, 2015). After JA responses are switched on, hormone signaling is attenuated by induction of the JA-responsive *JAZ* genes in order to avoid the inhibitory effect that over-activation of JA responses has on plant growth (Chini *et al.*, 2007; Thines *et al.*, 2007; Chung *et al.*, 2008; Zhang and Turner, 2008).

Exogenous application of JA has an inhibitory effect on primary root growth. Arabidopsis null mutants for positive JA signaling components develop longer roots in the presence of JA (Xie *et al.*, 1998; Chen *et al.*, 2011; Fernández-Calvo *et al.*, 2011).

Plants can modify their root system architecture (RSA) to avoid local high soil salt levels (Galvan-Ampudia and Testerink, 2011). During salt stress responses, regulatory transcriptional programs act together to control primary root growth through inhibition of cell division and elongation (West *et al.*, 2004; Geng *et al.*, 2013). Therefore, salt stress and activation of the JA pathway may have an inhibitory effect on primary root growth in Arabidopsis. In recent review articles, the JA pathway is linked to salt stress response (Kazan, 2015; Riemann *et al.*, 2015) under the following findings: (i) overexpression of the wheat JA-biosynthesis gene *OPR1* in Arabidopsis attenuates salt-mediated root growth inhibition (Dong *et al.*, 2013); (ii) rice (*Oryza sativa*) salt sensitive 3 (RSS3), a nuclear localized JAZ-interacting protein lacking a DNA binding domain, interacts with the OsbHLH089 and OsbHLH094 transcription factors forming a ternary complex that regulates salt-mediated root cell elongation in rice (Toda *et al.*, 2013); (iii) overexpression of the *OsCYP94C2b* gene encoding an OsCYP94C1-related enzyme that catalyzes the conversion of JA-Ile to an inactive form, enhances salt tolerance in rice (Kurotani *et al.*, 2015). This background suggests that JA signaling behaves as a positive or negative regulator of the salt stress response in a circumstantial manner (Riemann *et al.*, 2015). However, how the salt stress response can modulate the JA signaling pathway is not fully understood.

Large-scale transcriptomic studies have shown that some JA-biosynthesis genes (e.g. *AOC1*, *AOC2*, *AOS*, *LOX3* and *OPR3*) are up-regulated in roots under salt stress (Jiang and Deyholos, 2006; Ma *et al.*, 2006; Kilian *et al.*, 2007; Geng *et al.*, 2013). These findings suggest that the JA signaling pathway is activated by salt stress and that it triggers physiological and growth changes in plants. In 2013, Geng *et al.* described that the root growth rate in *jai3-1,* a JA-resistant mutant allele in Arabidopsis (encoding stabilized JAZ3), was higher than in wild-type plants under salt stress on a temporal basis. This finding is still to be confirmed in other well characterized JA-related mutants. Together, these results allow us to hypothesize that salt stress triggers activation of the JA signaling pathway in the roots leading to growth inhibition in Arabidopsis. However, the root zone where JA activation takes place remains to be identified, as are the cellular consequences of this activation on the primary growth of this plastic organ in direct contact with soil salt.

In this study, we show that salt stress triggers activation of the JA signaling pathway in a JAR1-, COI1- and proteasome-dependent manner in the meristematic zone and stele of the differentiation zone in the Arabidopsis root. This activation is likely to occur with the participation of core components of the JA signaling pathway, such as COI1, JAZ3 and MYC2/3/4, leading to inhibition of cell elongation in the primary root. All these findings indicate that the salt-stress response involves activation of the JA signaling pathway, resulting in inhibition of root growth in Arabidopsis.

## Materials and methods

### Plant materials and growth conditions

Wild-type Arabidopsis plants were of the Col-0 ecotype unless otherwise specified. Wild-type Col-6 seeds were obtained from ABRC (https://abrc.osu.edu, last accessed 9 May 2016). The *aos*, *coi1-1, coi1-2*, *jai3-1*, *jar1-1*, *myc2/3/4* and *35S::JAZ1-GUS* lines have been previously described (Xie *et al.*, 1998; Park *et al.*, 2002; Staswick and Tiryaki, 2004; Xiao *et al.*, 2004; Chini *et al.*, 2007; Thines *et al.*, 2007; Fernández-Calvo *et al.*, 2011) and listed in Supplementary Table S1.

*coi1-1* mutant segregants were identified in the progeny from the selfcrossed heterozygous *coi1-1* plants based on their root length after growing on MS medium supplemented with 50 μM MeJA (Sigma) for 4 d (Xie *et al.*, 1998).

Hydroponic seedlings used for qPCR assays and GUS staining were grown in PHYTATRAYS^®^ (Sigma) on a nylon filter (250 μm mesh) that is in direct contact with liquid 0.5× MS medium (Murashige and Skoog; Duchefa) supplemented with 0.5% (w/v) sucrose (Merck), 0.02% (w/v) MES (Sigma) and 1× Gamborg’s B-5 vitamin mixture (PhytoTechnology laboratories) as described (Kilian *et al.*, 2007). Plants were grown at 22 ^o^C under 16/8h light/dark cycles (120 μmol m^−2^ s^−1^) in a Percival culture chamber for 13 d. On day 13, liquid medium was replaced with sucrose free medium. The seedlings continued to grow until plant treatment on day 18. Seedlings used for root growth inhibition assays were grown on solid 1× MS medium containing 1% sucrose and 0.02% MES. Plates were placed vertically in a culture chamber and grown at 22 ^o^C for 5 d.

*35S::JAZ1-GUS* in *jar1-1* host genetic backgrounds were generated by crossing the corresponding homozygous parental plants. *35S::JAZ1-GUS* F_2_ segregating progenies of these crosses were selected based on their resistance to 50mg l^−1^ kanamycin and with a root growth inhibition assay using 50 μM MeJA and selecting for plants hyposensitive to the exogenous hormone (Staswick and Tiryaki, 2004). The reporter lines used for the histochemical GUS staining analysis came from the F_3_ progeny.

### Plant treatments

#### qPCR and GUS staining

Hydroponically grown 18-d-old plants were treated with 150mM NaCl (Kilian *et al.*, 2007), 10nM (*35S::JAZ1-GUS* line) or 50 μM JA as the methyl ester (MeJA) for 3h (RT-qPCR assays) or 6h (GUS staining assays).

#### Root growth inhibition assay

Five-day-old seedlings grown on solid medium were treated with 140mM NaCl for 18h and then five to 27 seedlings per genotype/treatment were analyzed.

#### 26S proteasome inhibition assay

*35S::JAZ1-GUS* and *35S::GUS* reporter lines were pretreated with 50 μM MG132^®^ (Sigma) in ethanol for 2h.

### Histochemical GUS staining

Seedlings were immersed in 90% acetone for 10min to stop treatment (Acosta *et al.*, 2013) and placed in GUS staining solution containing 100mM sodium phosphate buffer (Merck), pH 7.0, 10mM EDTA (Merck), 0.1% Triton X-100 (Calbiochem), 0.5mM potassium ferricyanide (Merck), 0.5mM potassium ferrocyanide (Merck), and 1.2mM 5-bromo-4-chloro-3-indolyl glucuronide (PhytoTechnology laboratories) as described in Jefferson *et al.*, 1987. Samples were incubated at 37 ^o^C for 15h. Tissue was cleared with 96% ethanol, rehydrated in water and mounted in 90% lactic acid on a slide. Observations were done under a stereoscopic light microscope (Olympus S261) and optical microscope (Olympus BY43).

### Time-lapse root imaging

Five-day-old seedlings grown vertically on solid medium were previously selected based on their root length homogeneity and transferred to solid 1× MS medium supplemented with or without 140mM NaCl. Root growth progression was registered starting at 3h, and then every 1h for a total time of 24h, using an automatic time lapse system with a 5-megapixel digital camera (Sony Cyber-shot DSC-F717) controlled by an intervalometer. Primary root growth images for each seedling were analyzed by the ImageJ software (http://imagej.nih.gov, last accessed 9 May 2016). Lengths in NaCl treated seedlings were normalized to the mock-treated control in each genotype.

### Root measurements

Five-day-old seedlings were treated with 140mM NaCl for 18h. Another group of seedlings were treated with 60mM LiCl (Shi *et al.*, 2002), 250mM mannitol (Liu *et al.*, 2014) or 10nM JA. The seedlings were immersed in 96% ethanol for 10min, rehydrated with distilled water and mounted in 90% lactic acid for observation under the microscope (Olympus BY43; Zeiss Primo Star). The length of the different root zones (DZ, differentiation zone; EZ, elongation zone; MZ, meristematic zone) were measured using ImageJ software. The MZ was delimited from the quiescent center (QC) to the first elongated cortical cell (Casamitjana-Martinez *et al.*, 2003). EZ was delimited from the first radicular hair protrusion to the outer apical portions of trichoblasts (Verbelen *et al.*, 2006). DZ extends from the EZ to the root-hypocotyl boundary.

### RNA isolation and cDNA synthesis

Total RNA was isolated from 75 roots of 18-d-old plants per genotype/treatment using the RNeasy Plant Mini Kit (Qiagen) and treated with RNase-free DNaseI (Zymo Research). First strand cDNA was synthesized from 2.5 μg of total RNA using SUPERSCRIPT^TM^ II reverse transcriptase (Invitrogen) according to the manufacturer’s instructions.

### Quantitative real-time RT-PCR analysis

The qPCR reactions were performed using Brilliant III Ultra-Fast SYBR^®^ Green qPCR Master Mix (Agilent Technologies) for dsDNA synthesis. For comparison of data from different PCR runs or cDNA samples, CT values for genes were normalized to the CT value of the reference gene *TIP41-like* (Livak and Schmittgen, 2001; Czechowski *et al.*, 2005; Gutierrez *et al.*, 2008) (Supplementary Fig. S1D). The relative transcript levels of the following genes (listed in Supplementary Table S2) were quantified: *JAZ1*, *JAZ2*, *JAZ3*, *JAZ4*, *JAZ5*, *JAZ6*, *JAZ7*, *JAZ8*, *JAZ9* and *MYC2*. Salt-responsive genes *CML37*, *PP2C* and *BCB* were used as positive controls (Gong *et al.*, 2001; Taji *et al.*, 2004; Kilian *et al.*, 2007; Vanderbeld and Snedden, 2007). All experiments were performed as three biological replicates and two technical replicates. The nucleotide sequences and efficiencies of the primer pairs used are listed in Supplementary Table S3. Thermal qPCR profile is described in Supplementary Table S4.

### Statistical analysis

Significant difference was calculated with pairwise Student’s *t*-test and multiple comparisons analysis were performed by ANOVA and Tukey’s tests using GRAPHPAD PRISM^®^ version 6.00 software (GraphPad Software, Inc.). Differences between mean values were considered to be significant with probability of 0.01% (****, *P*<0.0001), 0.1% (***, *P*<0.001), 1% (**, *P*<0.01) or 5% (*, *P*<0.05). Statistical significances between multiple treatments were indicated by a lowercase letter placed over the error bars.

## Results

### Salt stress induces JA-responsive *JAZ* gene expression in the roots by a COI1-dependent mechanism

**Fig. 1.**
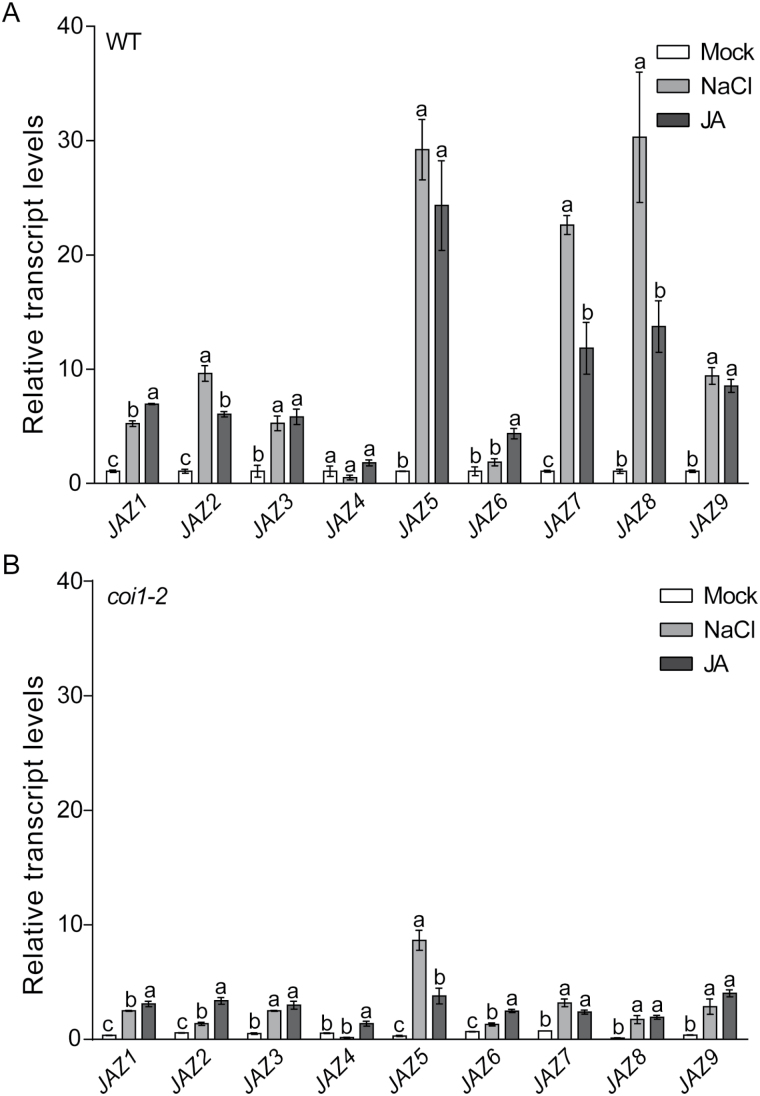
JA-responsive *JAZ* gene transcripts are up-regulated by salt stress in a COI1-dependent manner in Arabidopsis roots. (A) Relative expression of *JAZ* (1–9) in 18-d-old WT hydroponic plants under three different conditions: mock, 150mM NaCl or 50 µM JA treatment for 3h each. (B) Relative expression of *JAZ* (1–9) in *coi1-2* roots. *TIP41-like* was quantified by RT-qPCR and used as housekeeping gene. Transcripts levels in mock treatment for WT roots were arbitrarily set to one. Error bars show SEM of three independent experiments (trials) with two technical replicates. Multiple comparisons analysis was performed by ANOVA and Tukey’s tests. Statistical significances between treatments for each gene are shown by lowercase letters placed over the error bars.

Our initial target was to study whether JA-responsive genes are induced during the salt stress response in Arabidopsis roots. *JAZ*s are JA-responsive genes induced rapidly and strongly in a protein synthesis-independent manner by mechanical wounding or by the treatment of rosette leaves with exogenous JA (Chung *et al.*, 2008). Therefore, hydroponic 18-d-old plants were subjected to salt stress or JA treatment for 3h. The relative transcript levels of *JAZ* (1–9) genes were quantified in the roots by qPCR assays and compared to the mock control condition. As expected, salt treatment compared to non-treated plants resulted in increased (2.5- to 33-fold) transcript levels of three control salt responsive genes in the roots (Supplementary Fig. S1A–C). Importantly, all analyzed *JAZ* transcript levels were increased (5.2- to 30.3-fold) by salt stress in the roots, except for *JAZ4* and *JAZ6* whose transcripts levels were not significantly changed (Fig. 1A, Supplementary Table S5). On the other hand, *JAZ* transcript levels in the roots were increased (4.3- to 24.3-fold) by JA treatment, with the exception of *JAZ4* whose levels also did not significantly change (Fig. 1A, Supplementary Table S5). *JAZ* up-regulation by salt stress is consistent with the increased transcript levels visualized by eFP Browser (Winter *et al.*, 2007) abiotic stress series from microarray expression data for roots treated with NaCl (Kilian *et al.*, 2007) (Supplementary Table S5). These results suggest that salt stress triggers a strong activation of the JA signaling pathway in roots. We then analyzed the temporal dynamics of *JAZ* expression in root tissue from plants under salt stress using microarray expression data generated by Kilian *et al.* (2007). We found that there was a higher gene expression at 1h after the onset of salt treatment. They showed the highest peak of expression at 6h, and finally they were abruptly down-regulated 12h after the initiation of treatment (Supplementary Fig. S2A). These results suggest that JA signaling is activated and deactivated within a time frame of ~24h during the salt stress response. In addition, to study negative JA regulators like JAZ, we quantified the relative expression of *MYC2* (Dombrecht *et al.*, 2007) in the roots of seedlings under salt stress by qPCR (Supplementary Fig. S2B). We found that *MYC2* was up-regulated ~5-fold 3h after salt stress initiation. This result is supported by expression analysis of microarray public data sets (Kilian *et al.*, 2007) performed in our laboratory (Supplementary Fig. S2A). However, *COI1* expression measured by genome-wide gene expression analysis (Kilian *et al.*, 2007), was not up-regulated in the roots during salt treatment (Supplementary Fig. S2A). This result is consistent with variations in *COI1* expression during the salt stress response do not play a major role in the up-regulation of *JAZ* in the roots by salt treatment.

COI1-defective mutants are a useful tool for evaluating the contribution of JA to different biological processes (Browse, 2009). To study whether COI1 regulates salt-mediated up-regulation of *JAZ* genes, their transcript levels were quantified in the roots of *coi1-2* plants partially defective in JA-Ile perception (Xiao *et al.*, 2004) and compared with wild-type plants under salt stress (Fig. 1B). We found that *JAZ* transcript levels were in average 78.7% and 70.6% lower in *coi1-2* mutants than in wild-type roots under salt stress and JA treatment, respectively (Fig. 1B, Supplementary Table S6). These results indicate that COI1 function is required for salt-mediated up-regulation of *JAZ* in the roots.

### JAZ1 protein is destabilized by a proteasome- and JAR1-dependent mechanism in the roots in response to salt stress

**Fig. 2.**
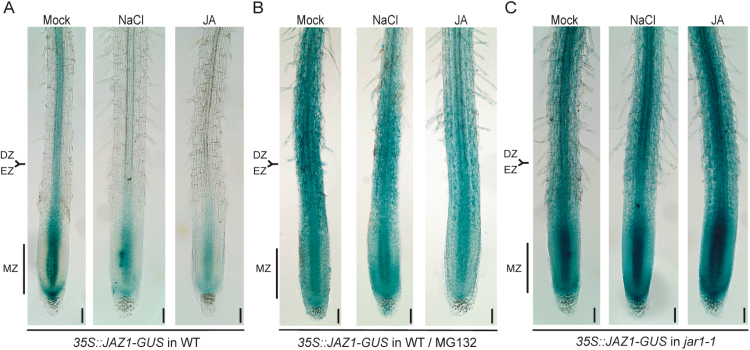
The JA-Ile sensor JAZ1-GUS is destabilized by salt stress in a JAR1 and proteasome-dependent manner in the roots. (A) Hydroponic 18-d-old plants expressing the JA-Ile sensor JAZ1-GUS from the *35S::JAZ1-GUS* construct were subjected to mock, 150mM NaCl or 10nM JA treatment for 6h and then stained for GUS activity. (B) As in panel A, with the difference that plants were preincubated with MG132 to inhibit proteasome activity before salt stress or JA treatment. (C) As in panel A, except that plants expressing *JAZ1-GUS* in *jar1-1* host genetic background were assayed. The photographs are representative of three independent experiments (trials) with eight biological replicates. DZ/EZ denotes the boundary region between the differentiation zone (DZ) and the elongation zone (EZ) while MZ indicates the meristematic zone. Scale bar, 100 μm. (This figure is available in colour at JXB online.)

To identify the root zone where the salt-mediated activation of JA signaling takes place we used *35S::JAZ1-GUS* plants expressing *JAZ1-GUS* driven by the CaMV 35S constitutive promoter (Thines *et al.*, 2007). The encoded fusion protein is destabilized by JA-Ile in a 26S proteasome-dependent manner in the roots (Thines *et al.*, 2007; Meesters *et al.*, 2014), acting therefore as a JA-Ile sensor for the endogenous bioactive JA-Ile (Fonseca *et al.*, 2009). 18-d-old hydroponic *35S::JAZ1-GUS* plants were subjected to salt stress or JA treatment for 6h and stained for GUS activity. We selected the 6h salt treatment based on the *JAZ* microarray expression data (Kilian *et al.*, 2007) analyzed in our laboratory. We observed that the highest up-regulation of *JAZ* genes takes place at this time point, with the exception of *JAZ4* that was not represented on the ATH1 GeneChip^®^ (Supplementary Fig. S2A). In *35S::JAZ1-GUS* plants under control conditions we observed GUS signal in the stele of primary roots, with a stronger staining in the differentiation zone (DZ), in the elongation zone (EZ) and in the center of the meristematic zone (MZ) (Fig. 2A). However, under salt or JA treatment we observed a significant reduction in GUS signal along the primary root of *35S::JAZ1-GUS* plants in the three zones already mentioned (DZ, EZ and MZ) when compared to the control condition (Fig. 2A). We further validated these results in plants of other developmental stages grown on solid medium (Supplementary Figs S3A, S4A).

The ubiquitin-proteasome pathway plays a major role in the activation of JA signaling via the ubiquitination of JAZs and their degradation by the 26S proteasome (Chini *et al.*, 2007; Thines *et al.*, 2007). To establish whether proteasome activity was necessary for the salt-mediated activation of JA signaling in the roots, 18-d-old *35S::JAZ1-GUS* hydroponic plants were pretreated with MG132 (Thines *et al.*, 2007; Meesters *et al.*, 2014), an inhibitor of proteasome activity, and then treated with salt or JA. We observed that JAZ1-GUS signal was partially stabilized by MG132 in salt and JA treatments when compared to non-MG132 pretreated seedlings, revealing that proteasome activity was necessary for activation of JA signaling during the salt stress response in the roots (Fig. 2A, B). We obtained similar results with 5-d-old seedlings grown on solid medium where a strong decrease in GUS signal in the center of the MZ (quiescent center) was observed after salt or JA treatment (Supplementary Fig. S3). However, this decrease was partially attenuated when seedlings were pretreated with MG132. Additionally, the protein levels of a constitutively expressed GUS in the *35S::GUS* line were not altered by salt, JA or MG132 treatment, showing that JAZ1-GUS levels are mostly dependent on JAZ1 stability (Supplementary Fig. S5). Together, these results show that during the salt stress response JAZ1 is destabilized by the proteasome, leading to activation of JA signaling in the roots.

To study whether an increase of JA-Ile levels is necessary for salt-stress mediated activation of JA signaling in the roots, the *35S::JAZ1-GUS* construct was introduced into the *jar1-1* host by crossing plants (Staswick and Tiryaki, 2004); then, homozygous segregants were stained for GUS activity. We found that the JAZ1-GUS destabilization triggered by salt or JA treatment in the wild-type host was abolished in 18-d-old (Fig. 2A, C) and 5-d-old *jar1-1* seedlings (Supplementary Fig. S6). These results show that JAR1 activity is required to activate JA signaling in the roots during the salt stress response.

### Inhibition of primary root growth mediated by salt stress is partially dependent on JA and occurs in the elongation zone

**Fig. 3.**
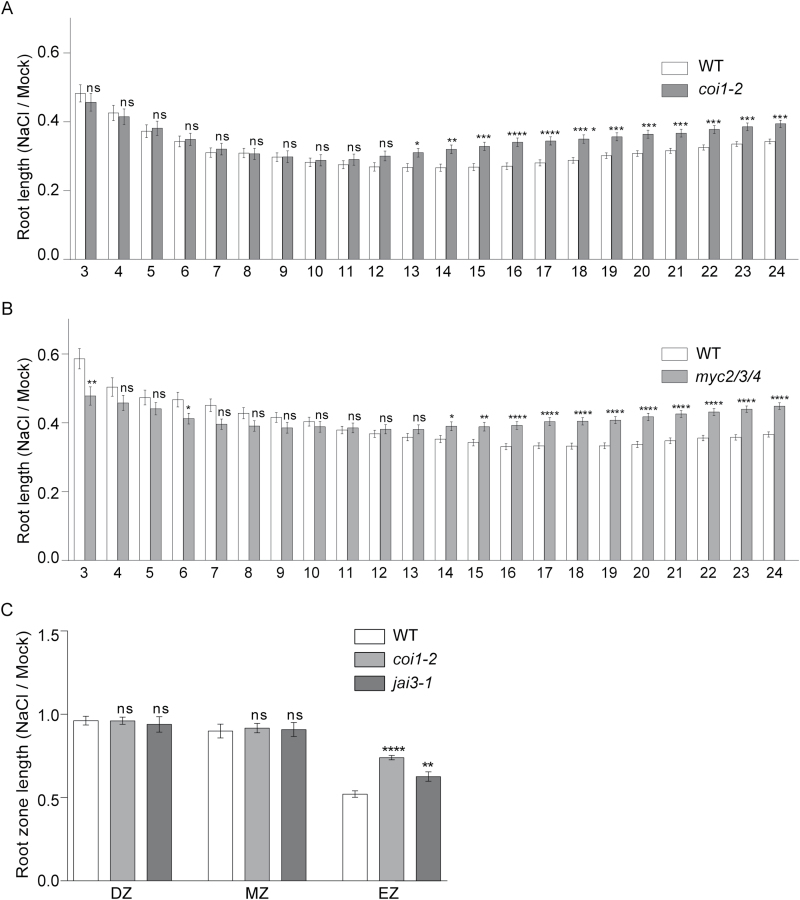
Salt-mediated inhibition of primary root growth is attenuated in JA signaling mutants. (A) 5-d-old WT and *coi1-2* seedlings grown on solid medium subjected to 140mM NaCl or mock treatment for 3–24h. During this period, the primary root was registered every 1h by an automatic time-lapse imaging system and the cumulative growth was plotted against time. (B) As in panel A, except that *myc2/3/4* seedlings were used. (C) WT, *coi1-2* and *jai3-1* seedlings grown on solid medium were subjected to NaCl or mock treatment for 18h and the differentiation zone (DZ), meristematic zone (MZ) and elongation zone (EZ) lengths were measured. The root measurements for NaCl treatment were normalized with the mock condition. These results are representative of three independent experiments (trials). Error bars represent SEM of biological replicates, *n*=27 (A, B) and *n*=15 (C); *, *P*<0.05; **, *P*<0.01; ***, *P*<0.001; ****, *P*<0.0001; ns, not significant, Student′s *t*-test.

**Fig. 4.**
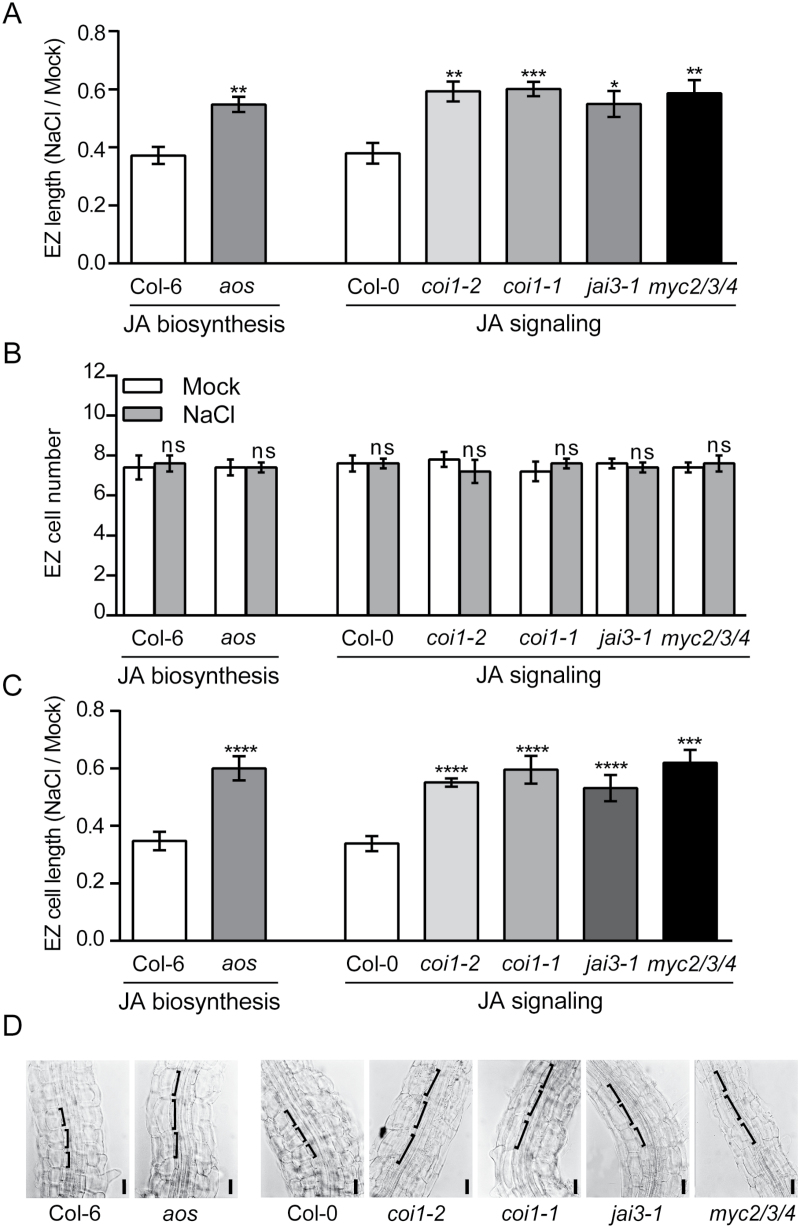
Salt-mediated inhibition of cell elongation in the EZ is attenuated in JA-related mutants. (A) 5-d-old *aos*, *coi1-2*, *coi1-1*, *jai3-1* and *myc2/3/4* mutants along with WT corresponding ecotypes (Col-6 and Col-0) grown on solid medium were subjected to 140mM NaCl or mock treatment for 18h. Elongation zone (EZ) length was measured. The root measurements for NaCl treatment were normalized with the mock condition. (B) As in panel A, except that cortical cells located in the EZ were counted. (C) As in panel A, except that cortical cell length in the EZ was measured. (D) Bright-field microscopy images of parts of the EZ. Square brackets show the longest dimension of cortical cells. Scale bar, 50 μm. These results are representative of three independent experiments (trials) with five biological replicates. Error bars represent SEM of biological replicates, *n*=5 (A, B) and *n*=24 (C); *, *P*<0.05; **, *P*<0.01; ***, *P*<0.001; ****, *P*<0.0001; ns, not significant, Student′s *t*-test.

Exogenous application of JA has an inhibitory effect on primary root growth. Mutants for JA-related signaling components such as COI1, JAZ3 and MYC2/3/4 develop longer primary roots in the presence of exogenous JA (Xie *et al.*, 1998; Chini *et al.*, 2007; Fernández-Calvo *et al.*, 2011). Therefore, we speculated whether the activation of JA signaling plays an inhibitory role on primary root growth under salt stress. Thus, we performed a root growth inhibition assay using an automatic time-lapse imaging system to compare the root growth response of *coi1-2* and *myc2/3/4* (*myc2 myc3 myc4*) plants with wild-type counterparts every hour during salt treatments lasting 3–24h (Fig. 3A, B). For the treatment of seedlings we used 140mM NaCl as this salt concentration inhibits root growth but is non-lethal (Geng *et al.*, 2013). We observed that root growth was inhibited by salt stress in *coi1-2*, *myc2/3/4* and wild-type plants. The lengths of *coi1-2* and *myc2/3/4* primary roots were significantly higher than those of the WT under salt treatment, with a maximum significant difference shared among mutants at 16–20h, with longer roots (18–25%) than WT (Fig. 3A, B). In order to determine the root zone where growth was inhibited by salt stress in a JA-dependent manner, we measured the length of the DZ, MZ and EZ in wild-type, *coi1-2* and *jai3-1* seedlings under salt treatment for 18h (Fig. 3C). The relative length of the EZ (but not the DZ or MZ) in wild-type seedlings was greatly reduced by salt treatment (Fig. 3C). Interestingly, the relative EZ in *coi1-2* and *jai3-1* were longer by 42% and 22%, respectively, when compared with wild-type plants after 18h salt treatment, indicating that salt stress inhibits EZ growth by a mechanism that is partially dependent on COI1 and JAZ3 (Fig. 3C). Similar results were obtained with JA-biosynthesis (*aos*) and JA-signaling mutants such as *coi1-1* and *myc2/3/4* under salt stress for 18h (Fig. 4A). Their EZs were on average 52% longer than those of wild-type plants, indicating that these JA-related mutants have an attenuated salt-mediated inhibition of EZ growth. Together, these results show that there is a JA-dependent inhibition of EZ growth 18h after salt stress begins.

### Salt stress triggers a JA-dependent inhibition of cell elongation in the EZ

The salt-mediated and JA-dependent root growth inhibition in the EZ could be the consequence of changes in the total number of cells and/or their size within the EZ. To discriminate between these possibilities, we counted cortical cells located in the EZ of *aos*, *coi1-1, coi1-2*, *jai3-1*, *myc2/3/4* and wild-type seedlings after 18h of salt treatment. No significant differences in cell number were observed between the mock and salt treatment of JA-related mutants and wild-type EZs (Fig. 4B). We measured the length of cortical cells located in the EZ of JA-related mutants and wild-type seedlings after 18h of salt treatment. We found that the relative length of EZ cells in *aos*, *coi1-1, coi1-2*, *jai3-1* and *myc2/3/4* was an average 70% higher than wild-type counterparts during 18h salt treatment (Fig. 4C, D). These results suggest that JA biosynthesis as well as the canonical components of JA signaling are partially responsible for a salt-mediated inhibition of cell elongation in the EZ.

*coi1-1* is a well-characterized null mutant with a fully impaired JA-Ile signaling pathway (Xie *et al.*, 1998). Interestingly, we observed that salt-mediated inhibition of EZ cell elongation was attenuated ~23% in the *coi1-1* mutant compared to wild-type after 18h of salt treatment (Table 1). These results suggest the existence of JA-dependent and JA-independent signaling pathways involved in the inhibition of cell elongation in the EZ during the salt stress response.

**Table 1.** Comparison of lengths of cortical EZ cells from JA-related mutants and WT seedlings under salt stress

**Genotype**	**Ecotype**	**Length Mock**	**Length** **NaCl**	**Inhibition (%**)	*P*-value*
WT	Col-0	90.2±6.7	33.7±2.3	62.6	-
*coi1-2*	Col-0	95.4±9.2	45.1±1.2	52.7	<0.00001
*coi1-1*	Col-0	80.1±5.2	48.2±4.1	39.8	<0.001
*myc2/3/4*	Col-0	78.9±5.9	49.2±3.5	37.6	<0.0001
*jai3-1*	Col-0	106.5±8.8	53.2±4.6	50.0	<0.00001
WT	Col-6	96.5±7.3	33.5±3.1	65.3	-
*aos*	Col-6	85.2±8.1	46.2±3.6	45.8	<0.0001

5-d-old WT and JA-related mutant seedlings grown on solid medium subjected to 140mM NaCl or mock treatment during 18h. The cell length values (µm) are mean±SEM of cortical cells in the elongation zone (EZ) per genotype (*n*=24). Cell growth inhibition (%) by salt stress was calculated from the length ratio between salt and mock treatments per each genotype. * Significance of median for ratios of the length (salt/mock) between mutant and WT plants.

### Osmotic stress inhibits cell elongation in a COI1-dependent manner

**Fig. 5.**
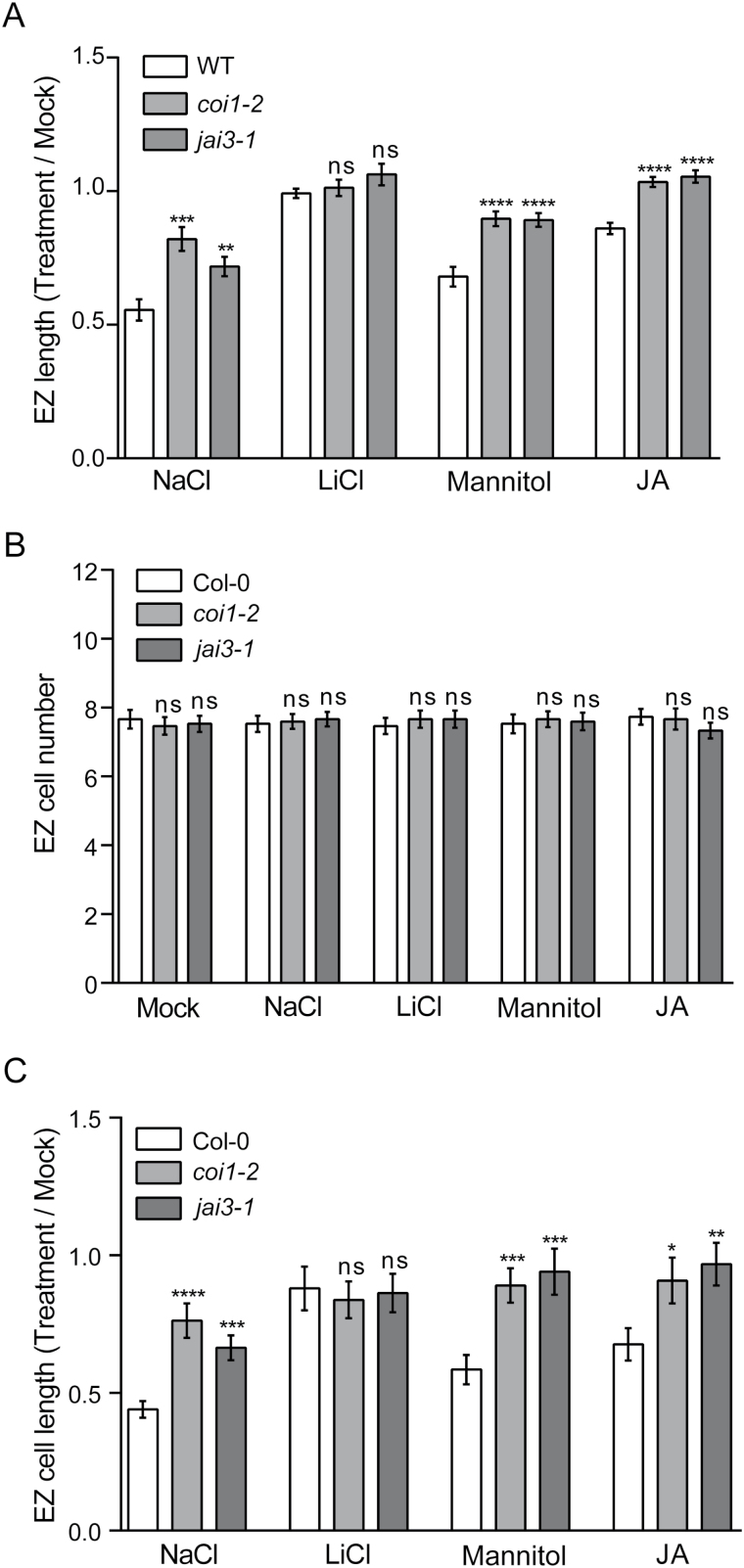
Osmotic stress inhibits cell elongation in the EZ in a COI1 and JAZ3-dependent manner. (A) 5-d-old WT, *coi1-2* and *jai3-1* seedlings grown on solid medium were subjected to 140mM NaCl, 60mM LiCl, 250mM mannitol or 10nM JA or mock treatment for 18h and then the EZ length was measured. (B) As in panel A, except that cortical cells located in the EZ were counted. (C) As in panel A, except that the cell length of cortical cells in the EZ was measured. The root measurements for all treatments were normalized with the mock condition, except for cell number determinations. These results are representative of three independent experiments (trials). Error bars represent SEM of biological replicates, *n*=15; *, *P*<0.05; **, *P*<0.01; ***, *P*<0.001; ****, *P*<0.001; ns, not significant, Student′s *t*-test.

Salt-stress mediated by NaCl has two components: ionic stress and osmotic stress (Zhu, 2002). Therefore, we decided to establish what salt-stress component plays a role in the JA-dependent growth inhibition of the EZ. In order to accomplish this goal, 5-d-old *coi1-2*, *jai3-1* and wild-type seedlings were treated with NaCl, LiCl (ionic stress), mannitol (osmotic stress) or JA for 18h. Lithium is the sodium analog with higher toxicity able to create ionic stress in plants and yeast with a significant lower doses than sodium and reduced contribution to osmotic stress (Shi *et al.*, 2002; Rausell *et al.*, 2003). We measured the length of the EZ (Fig. 5A) and observed significantly shorter EZs (14–44%) in wild-type seedlings treated with NaCl, mannitol or JA compared to the non-treated counterparts, indicating that EZ growth was inhibited in all of them (Fig. 5A). However, the EZ length of wild-type seedlings treated with LiCl was similar to that of non-treated plants, showing that ionic stress by itself is not able to inhibit growth in the EZ (Fig. 5A). The growth inhibition mediated by mannitol observed in the EZ of wild-type plants was significantly reduced in *coi1*-2 and *jai3-1* plants, indicating that osmotic stress-mediated growth inhibition in this zone is dependent on COI1 and JAZ3 functions (Fig. 5A). Cortical cell number in the EZ was not altered in *coi1-2*, *jai3-1* and wild-type seedlings under the same treatments described above (Fig. 5B). We measured the relative length of cortical cells in the EZ of *coi1-2*, *jai3-1* and wild-type seedlings treated with NaCl, LiCl, mannitol or JA for 18h. We found that mannitol-mediated growth inhibition of cortical cells in the EZ was higher in wild-type plants (42%) compared to 11% and 6% in *coi1-2* and *jai3*-1 plants, respectively (Fig. 5C). However, the cell length of EZ cells was not significantly altered by LiCl treatment. These results suggest that ionic stress is not playing by itself a major role in salt-mediated and JA-dependent cell elongation inhibition in the EZ. As expected, JA-mediated inhibition of cell elongation in the EZ was higher in wild-type plants (32% compared to 8% and 4% in *coi1-2* and *jai3*-1 plants, respectively (Fig. 5C). Therefore, these results show that the osmotic component of salt stress inhibits cell elongation in the EZ by a COI1-dependent mechanism.

## Discussion

### *JAZ* up-regulation in the roots reveals activation of JA signaling during the salt stress response

Previous large-scale expression studies showed that some genes implicated in JA biosynthesis were induced in the roots by salt stress, suggesting that the biosynthesis of this hormone is induced in roots by salt stress (Jiang and Deyholos, 2006; Ma *et al.*, 2006; Kilian *et al.*, 2007). In order to gain insight into early JA-responsive gene activation in the roots during the salt stress response, we quantified *JAZ* expression in plants and compared with microarray expression data sets by Kilian *et al.* (2007) (Fig. 1, Supplementary Table S5). Interestingly, the first study was repeatable using a different approach to quantify transcript levels. We found that eight out of nine analyzed *JAZ* genes are up-regulated by salt stress. Together, these gene expression results strongly suggest that JA signaling is activated in the roots at early stages of the salt stress response. Since JAZ proteins are negative regulators of JA signaling, it was proposed that their gene up-regulation by wounding or exogenous application of JA in the vegetative tissue may constitute a negative-feedback mechanism to turn off hormone signaling once the signal has been transmitted (Chini *et al.*, 2007; Thines *et al.*, 2007; Chung *et al.*, 2008). Therefore, the *JAZ* up-regulation observed in the roots after 3h of salt treatment suggests that JAZ proteins are ‘on their way’ to turn off the JA pathway. Interestingly, *JAZ* expression levels were partially reduced in *coi1-2* (Xiao *et al.*, 2004) roots compared with wild-type plants under salt stress (Fig. 1B, Supplementary Table S6). This result shows that COI1, a core component of the JA-Ile co-receptor (Sheard *et al.*, 2010), is necessary for *JAZ* transcript up-regulation in the roots during the response to salt stress.

### Salt stress triggers JAZ1 destabilization in the roots by a JAR1-dependent mechanism

The salt-mediated and COI1-dependent up-regulation of *JAZ* genes observed in the roots (presented here) is likely to follow the canonical JA signaling pathway (Wasternack and Hause, 2013), with proteasome-dependent degradation of JAZ proteins. These assumptions were further confirmed by the visualization of JAZ1 destabilization in root tissues using the JA-Ile sensor JAZ1-GUS line. This assay showed substantial activation of JA signaling after salt or JA treatment, mainly in the stele and lower part of the MZ by a JAR1-dependent mechanism and involves the proteasome-mediated destabilization of JAZ1 (Fig. 2). Therefore, salt-mediated activation of JA signaling in the roots involves the canonical components of the JA signaling pathway. The origin of signal from roots grown under salt stress and how it is transduced and integrated from different upstream signals into up-regulation of JA-Ile biosynthesis or down-regulation of hormone catabolism is not understood. Further studies are necessary to elucidate early events of JA-Ile homeostasis in the roots.

Recently, Geng *et al.* (2013) reported a high-resolution spatiotemporal transcriptional map for the root response to salt stress in Arabidopsis. We compared the root zone patterns for activation of JA signaling (using the JA-Ile sensor line) with the spatiotemporal transcriptional maps of *JAZ5*, *JAZ7* and *JAZ8,* which were highly (23–30-fold) up-regulated in the roots during the salt stress response (Supplementary Table S5). We found that activation of JA signaling in the MZ and stele of the DZ correlated with *JAZ5* up-regulation in the stele during 8h of salt treatment on a spatiotemporal transcriptional map (Supplementary Fig. S4). For the case of *JAZ7* and *JAZ8*, this up-regulation takes place preferentially in cortical cells, indicating that JA signaling is activated in this cell layer during 8h of salt treatment. This activation occurs before the salt-mediated and JA-dependent cell elongation inhibition in cortical cells of the EZ occurring no later than 18h of salt treatment (Fig. 4C). JAZ8 is a stabilized protein against JA-mediated degradation while JAZ5, JAZ6 and JAZ7 are predicted to be stable because they also lack a canonical degron required for efficient COI1 binding in a JA-Ile dependent manner (Shyu *et al.*, 2012). These data suggest that EAR-motif based JAZ5, JAZ7 and JAZ8 repressors bind to MYC2/3/4 due to their higher abundance in the presence of JA-Ile, repressing JA-dependent expression (Shyu *et al.*, 2012). Based on this data, we can hypothesize that salt-mediated up-regulation of *JAZ5*, *JAZ7* and *JAZ8* establishes a negative feedback loop that turns off JA signaling to attain a rapid growth recovery phase during root adaptation to salt stress.

### Salt stress triggers the inhibition of cell elongation in the EZ in a JA-dependent manner

**Fig. 6.**
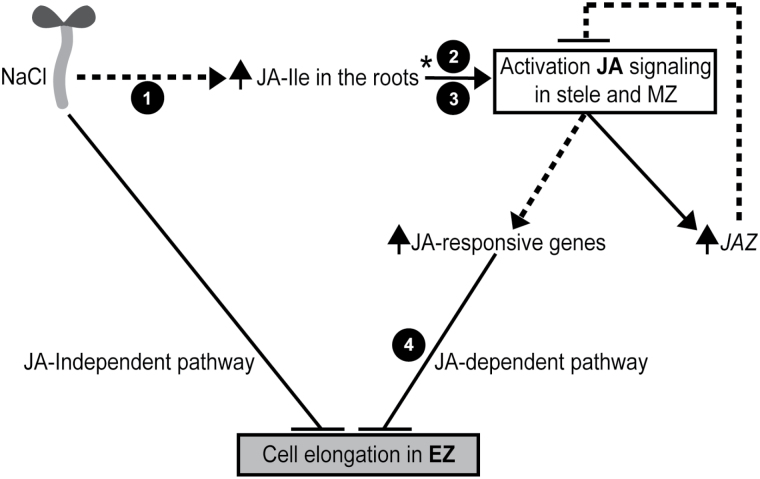
A model for salt-mediated and JA-dependent cell elongation inhibition in Arabidopsis roots. Salt-mediated cell elongation inhibition could be regulated by two pathways. The first is JA independent while the second one is JA dependent. The JA-dependent pathway probably begins with an increase of JA-Ile levels in the MZ and the stele (DZ) by a still unknown mechanism leading to JA signaling activation (this work). Once JA signaling is activated the up-regulation of JA-responsive genes such as *JAZ* (this work) and others (presumed) occurs. During salt stress response cell elongation is inhibited in the EZ (this work) decreasing primary root growth in Arabidopsis. The dashed lines indicate presumed steps of the pathway. The numbers 1, 2 and 3 indicate steps dependent on JAR1, COI1 and proteasome, respectively. Cell elongation inhibition in the EZ represented by number 4, is triggered by salt stress or osmotic stress by a mechanism dependent on AOS, COI1, JAZ3 destabilization and MYC2/3/4. The asterisk denotes a presumed increase of JA-Ile levels based on JAZ1-GUS stability in the JA-Ile sensor line.

During early stages of the salt stress response, the effects of NaCl are governed by its osmotic component, which affects water availability in plants inhibiting cell elongation (Geng *et al.*, 2013; Julkowska *et al.*, 2014). Our results show that there is a salt-mediated activation of JA signaling but, in contrast, it is well known that NaCl or JA application has an inhibitory effect on root growth (Xie *et al.*, 1998; Zhu *et al.*, 1998). We found significant differences in cell elongation in the EZ between JA-related mutants and wild-type seedlings under salt stress (Fig. 4C). Therefore, the activation of JA signaling during the salt stress response leads to inhibition of cell elongation in the EZ, albeit confined to a specific root zone. It is important to mention that Geng *et al.* (2013), using a high resolution time-lapse imaging system, showed that the JA insensitive *jai3-1* mutant (Chini *et al.*, 2007), which contains a mutation in the *JAZ3* gene rendering a stabilized protein, exhibits increased primary root growth rate compared with wild-type plants during short salt treatments. Our results are consistent with this previous study, showing that the destabilization of the JAZ3 repressor plays an important role in the salt-mediated and JA-dependent root growth inhibition, occurring as cell elongation inhibition in the EZ during the early response to salt stress. Furthermore, we have demonstrated that osmotic (mannitol) stress can partially mimic the salt-mediated and JA-dependent inhibitory effect on EZ cell elongation (Fig. 5C). A rapid increase in soil salinity causes leaf cells to lose water with a decreasing turgor (Munns and Tester, 2008). We could infer that activation of JA signaling during the salt stress response could be the consequence of decreased water availability and loss in turgency, although further studies are necessary to verify this. However, the salt-mediated cell elongation inhibition in *coi1-1* EZ was still present when plants were under salt stress for 18h. This result suggests the existence of an alternative pathway for salt-mediated growth inhibition in EZ that is independent of JA signaling (Figs 5C, 6). Exogenous JA reduces meristem cell number via MYC2 during long treatment times; but this hormone has also an inhibitory effect on DZ and EZ length, probably not completely fulfilled by MYC2 function (Chen *et al.*, 2011). Besides, it has been reported that JA hypersensitive *ninja* mutants have reduced cell elongation in the DZ compared to wild-type plants and that this phenotype was not suppressed in a *ninja myc2* double mutant (Acosta *et al.*, 2013). These results indicate that other or additional transcription factors are being repressed by NINJA in the roots. Therefore, further studies are needed because it is not well understood how JA inhibits cell elongation in the DZ and EZ at a molecular and cellular level. Interestingly, we found that the inhibition of cell elongation in the EZ at 18h of salt treatment was significantly attenuated in the *myc2/3/4* mutant compared to wild-type plants, similar to other JA-related mutants (Fig. 4B, C). These results show that MYC2/3/4 play an important role in salt-mediated JA-dependent inhibition of cell elongation in the EZ.

Using the *aos* (Park *et al.*, 2002) mutant, defective in the biosynthesis of jasmonates, we showed that part of the salt-mediated inhibition of cell elongation in the EZ takes place by a mechanism requiring JA (Fig. 4C). However, cyclopentanone 12-oxo-phytodienoic acid (OPDA), a precursor of JA biosynthesis, or other JA derivatives cannot be ruled out as possible signaling molecules involved in salt-dependent inhibition of cell elongation in the EZ. It was recently shown that exogenous application of OPDA inhibits primary root growth by a COI1-dependent mechanism (Mueller *et al.*, 2008; Stotz *et al.*, 2013) suggesting that OPDA or its conversion to JA-Ile is playing a relevant role in OPDA-mediated root growth inhibition. These results raise the question whether salt-mediated inhibition of EZ cell elongation is mediated directly by OPDA or JA-Ile. However, we found that activation of JA signaling in the roots during the salt stress response visualized by JA-Ile biosensor is dependent on JAR1 activity suggesting that JA-Ile is playing a role in this process (Fig. 2C, Supplementary Fig. S6B). Moreover, we observed that in JA-signaling mutants such as *coi1-1*, *jai3-1* and *myc2/3/4* the salt-mediated inhibition of cell elongation is attenuated with respect to wild-type plants (Fig. 4C). Together, these effects insinuate that components of the canonical JA-Ile signaling are playing an active role and OPDA alone likely is not totally involved in salt-mediated root growth inhibition.

### Comparison of salt stress-triggered JA-dependent inhibition of cell elongation between Arabidopsis and rice

Rice salt-sensitive 3 (RSS3) is a recently described JAZ-interacting protein that modulates salt-mediated inhibition of cell elongation in rice roots (Toda *et al.*, 2013). RSS3 interacts with two members of the bHLH family of transcription factors forming a ternary complex with OsJAZ9 or OsJAZ11 that negatively regulates JA-responsive genes in the roots. Interestingly, we observed some similarities between the salt-JA relationship described by Toda *et al.* (2013) in rice and that in Arabidopsis roots (this work). The salt-mediated inhibition of cell elongation observed in *rss3*, a JA-hypersensitive mutant, is enhanced compared to wild-type in rice (Toda *et al.*, 2013) while in Arabidopsis JA-hyposensitive mutants it is attenuated (presented here). Collectively, this proposes that salt-mediated JA signaling activation leading to inhibition of cell elongation is conserved in at least two glycophytes, including model systems for monocots and dicots.

### Integrated model for salt stress-triggered activation of JA signaling leading to inhibition of cell elongation in the EZ

Based on the fact that inhibition of cell elongation in the EZ during the salt stress response is still observed in a fully JA insensitive *coi1-1* (Xie *et al.*, 1998) mutant (Fig. 4C, Table 1), we propose two possible salt-mediated pathways that lead to root growth inhibition in Arabidopsis (Fig. 6). The first pathway is JA independent, while the second is triggered by osmotic stress and is mediated by components of the canonical JA signaling pathway.

The JA-dependent pathway probably begins either with a salt-dependent increase of JA-Ile levels in the MZ and the stele (DZ) through JA-Ile biosynthesis dependent on JAR1 or inhibition of JA-Ile turnover. Salt has a still unknown transduction mechanism that raises JA-Ile levels in roots as described earlier. Then, high local levels of JA-Ile lead to activation of JA signaling in these root regions.

Once JA signaling is activated, there is an up-regulation of JA-responsive genes, such as *JAZ*, among other unknown genes leading later on to the inhibition of cell elongation in the EZ, with consequences on root growth.

It is important to mention that there is no evidence supporting the idea that JA-dependent root growth inhibition triggered by salt is playing a role in plant tolerance to this abiotic stress. There are a few examples where a relationship between JA and salt tolerance has been recently documented (Kazan, 2015; Riemann *et al.*, 2015). Further studies would be necessary to address whether JA-dependent and salt-mediated root growth inhibition plays a relevant role to plant surviving or adapting to salt stress.

Taken together, our results showed that the inhibitory effect of salinity on root growth partially involves the jasmonate signaling pathway in Arabidopsis.

## Supplementary data

Supplementary data are available at *JXB* online.

Fig. S1. Relative transcript levels of control salt-induced genes in roots under salt stress.

Fig. S2. Expression levels of JA-responsive genes in the roots at different time points under salt stress.

Fig. S3. The JA-Ile sensor JAZ1-GUS is destabilized by salt stress in the roots in a proteasome-dependent manner.

Fig. S4. Activation of JA signaling and spatiotemporal *JAZ* expression in the roots during the salt stress response.

Fig. S5. GUS protein levels are not affected by salt stress or by inhibition of proteasome activity in the roots.

Fig. S6. The JA-Ile sensor JAZ1-GUS is destabilized by salt stress in the roots in a JAR1-dependent manner.

Table S1. JA-related mutants used in this study.

Table S2. AGI locus identifiers with gene description.

Table S3. Nucleotide sequence of the set of primers used in qPCR assays.

Table S4. qPCR thermal cycle profile used for all genes assayed.

Table S5. Comparison of *JAZ* genes expression in Arabidopsis roots under salt or JA treatment.

Table S6. Comparison of *JAZ* genes expression in WT and *coi1-2* roots under salt or JA treatment.

Supplementary Data

## Abbreviations:

AOS: allene oxide synthase
COI1: coronatine insensitive 1
DZ: differentiation zone
EZ: elongation zone
GUS: β-glucoronidase
JA: jasmonate
JA-Ile: jasmonoyl-isoleucine
*jai3-1*: *jasmonate-insensitive 3-1*
JAR1: jasmonate resistant 1
JAZ: jasmonate-ZIM domain
MS: Murashige and Skoog
*myc2/3/4*: *myc2 myc3 myc4*
MZ: meristematic zone
WT: wild type.
