# Supplementary material for: Salt stress response triggers activation of the jasmonate signaling pathway leading to inhibition of cell elongation in Arabidopsis primary root
Source: J Exp Bot. 2016 May 23;67(14):4209–20. doi: 10.1093/jxb/erw202 (PMC5301928; doi:10.1093/jxb/erw202)
Supplement: Supplementary Data [file supp_67_14_4209__index.html]

Salt stress response triggers activation of the jasmonate signaling pathway leading to inhibition of cell elongation in Arabidopsis primary root — Salt stress response triggers activation of the jasmonate signaling pathway leading to inhibition of cell elongation in Arabidopsis primary root — Supplementary Data 

# Salt stress response triggers activation of the jasmonate signaling pathway leading to inhibition of cell elongation in Arabidopsis primary root

## Supplementary Data

Data files

- supplementary\_figures\_S1\_S6\_Tables\_S1\_S6.pdf - Supplementary Data
