# Supplementary material for: Salt stress response triggers activation of the jasmonate signaling pathway leading to inhibition of cell elongation in Arabidopsis primary root
Source: J Exp Bot. 2016 May 23;67(14):4209–20. doi: 10.1093/jxb/erw202 (PMC5301928; doi:10.1093/jxb/erw202)
Supplement: Supplementary Data [file supp_erw202_supplementary_figures_S1_S6_Tables_S1_S6.pdf]

Supplementary data

Salt stress response triggers activation of the jasmonate signaling pathway leading to inhibition of cell elongation in Arabidopsis primary root

Camilo E. Valenzuela, Orlando Acevedo-Acevedo, Giovanna S. Miranda, Pablo Vergara-Barros, Loreto Holuigue, Carlos R. Figueroa and Pablo M. Figueroa*

*Author for correspondence: Pablo M. Figueroa (E-mail: pabfigueroa@utalca.cl)
Instituto de Ciencias Biológicas, Universidad de Talca, Talca 3465548, Chile

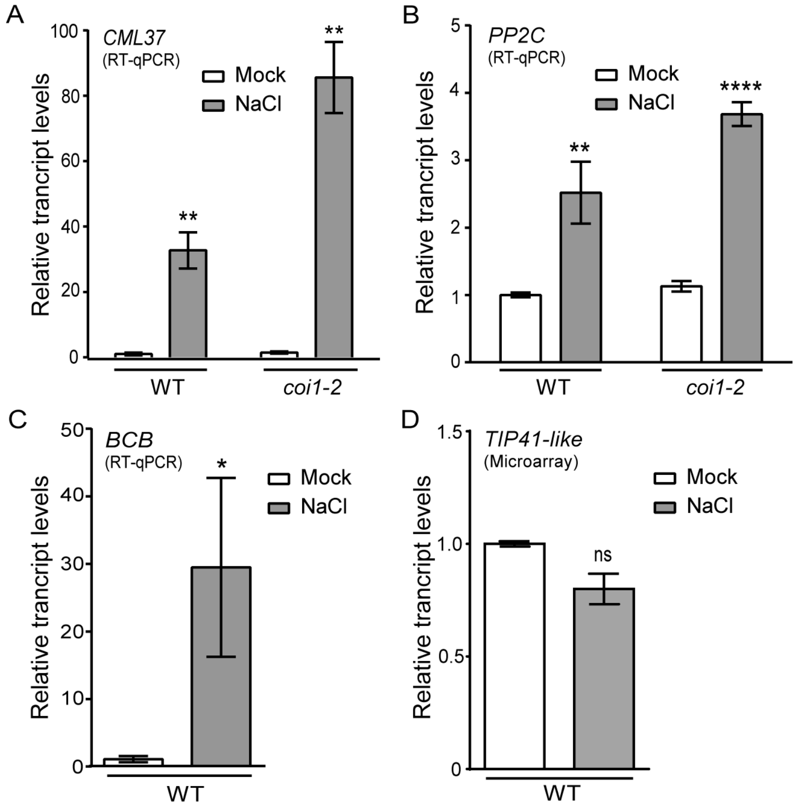

Supplementary Fig. S1. Relative transcript levels of control salt-induced genes in roots under salt stress.

(A) *CML37* (At5g42380) and (B) *PP2C* (At3g05640) transcript levels in the roots from 18-d-old WT and *coi1-2* plants under mock or 150 mM NaCl treatment for 3h were quantified by RT-qPCR assays. (C) *BCB* (At5g20230) transcript levels in WT roots were quantified. (D) *TIP41-like* (At4g34270) transcript levels in the roots from 18-d-old WT plants quantified by microarray analysis (Kilian *et al.*, 2007). *TIP41-like* was used as a housekeeping gene (Czechowski *et al.*, 2005; Gutierrez *et al.*, 2008) for the RT-qPCR assays shown in this work. Error bars represent SEM of three biological replicates with two technical replicates (A to C) and two biological replicates (D). *P<0.05; **P<0.01; ****P<0.0001; ns, not significant, Student's t-test.

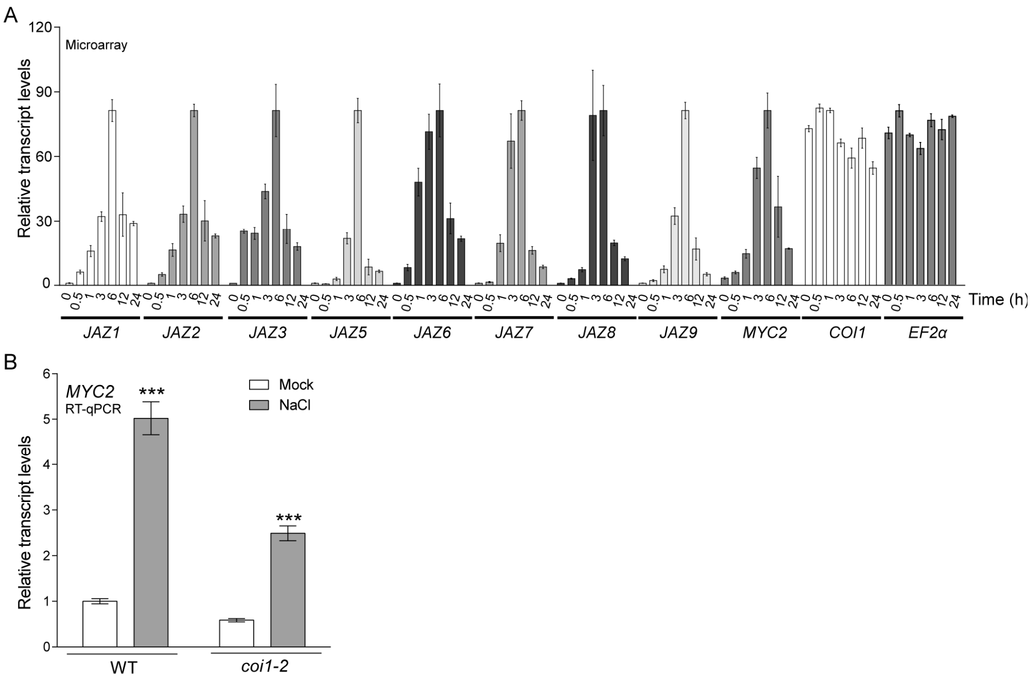

Supplementary Fig. S2. Expression levels of JA-responsive genes in the roots at different time point during salt stress.

(A) *JAZ1* (At1g19180), *JAZ2* (At1g74950), *JAZ3* (At3g17860), *JAZ5* (At1g17380), *JAZ6* (At1g72450), *JAZ7* (At2g34600), *JAZ8* (At1g30135), *JAZ9* (At1g70700), *MYC2* (At1g32640), *COI1* (At2g39940), and *EF2α* (At5g60390) transcript levels in the roots from 18-d-old WT plants under 150 mM NaCl treatment quentified at different time points by microarray analysis (Kilian *et al.*, 2007). (B) *MYC2* (At1g32640) transcript levels in the roots from 18-d-old WT and *coi1-2* plants under 150 mM NaCl treatment for 3h quantified by RT-qPCR assays. For microarray analysis the transcript levels in mock treatment was arbitrarily set to one. *EF2α* is a housekeeping gene. Error bars represent SEM of two biological replicates for microarray analysis (A) and three biological replicates with two technical replicates for RT-qPCR assays (B). ***$P<0.001$, Student's t-test.

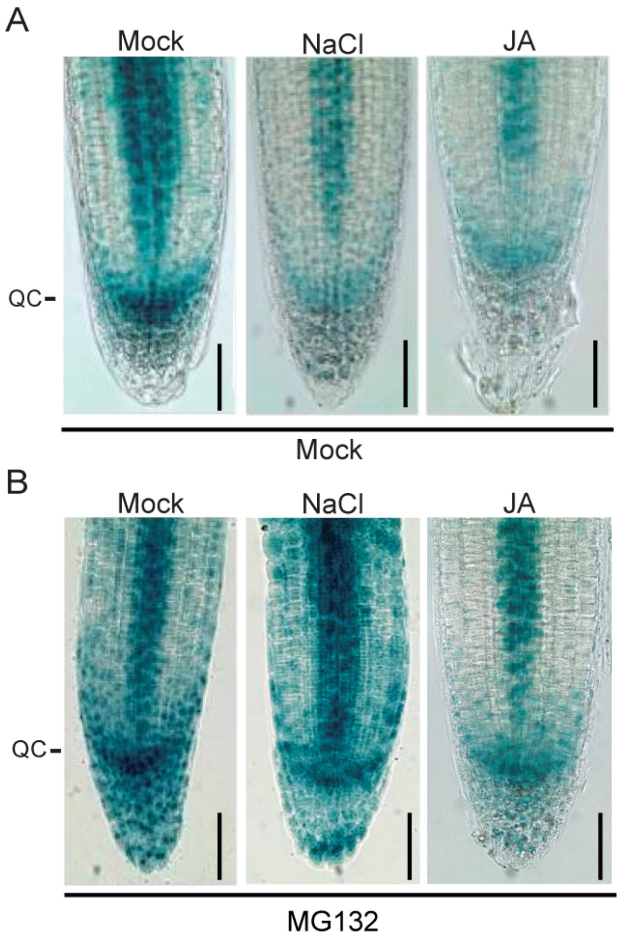

Supplementary Fig. S3. The JA-Ile sensor JAZ1-GUS is destabilized by salt stress in the roots in a proteasome dependent manner.
(A) 5-d-old seedlings roots expressing the JA-Ile sensor JAZ1-GUS were subjected to mock, 150 mM NaCl or 10 nM JA treatment for 6h and stained for GUS activity. (B) Proteosome activity was inhibited before treatment by preincubating roots with 50 μM MG132 for 2h. (A) and (B) are representative of three independent experiments with five biological replicates. QC denotes quiescent center. Scale bar 50 μm.

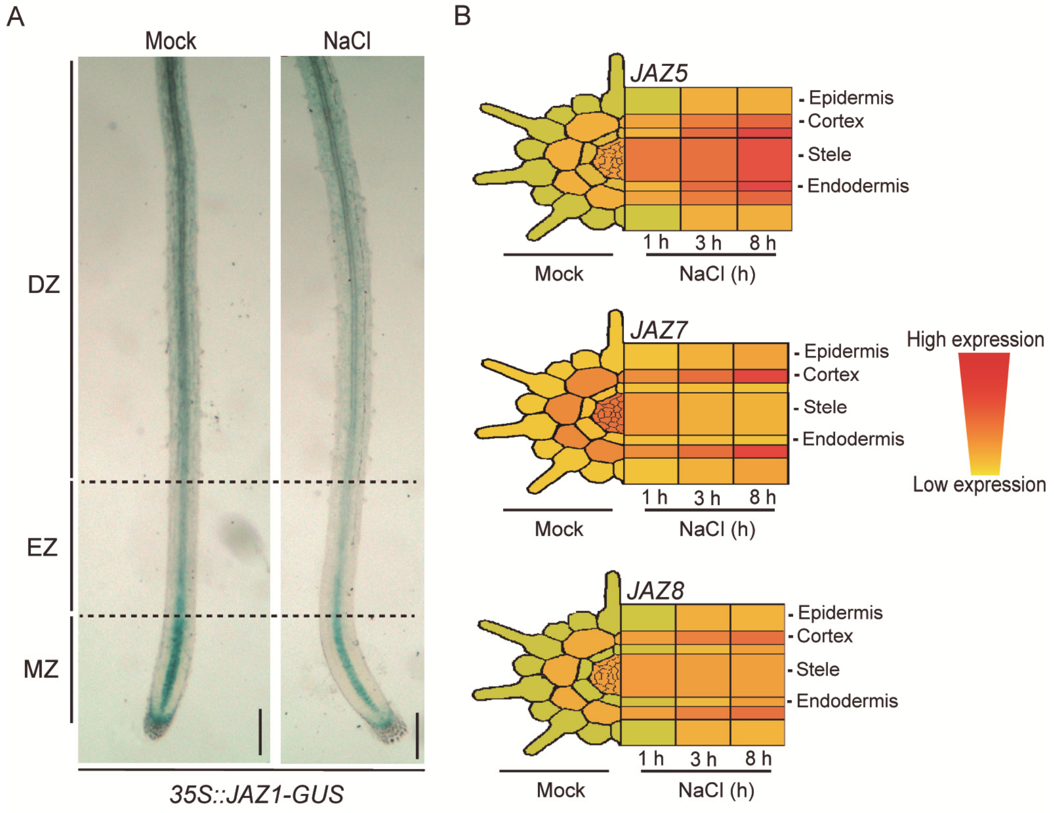

Supplementary Fig. S4. Activation of JA signaling and spatiotemporal *JAZ* expression in the roots during the salt stress response.

(A) 5-d-old seedlings expressing the JA-Ile sensor JAZ1-GUS subjected to mock or 140 mM NaCl treatment for 6h and stained for GUS activity. Scale bar 200 μm. (B) 5-d-old seedlings subjected to mock or 140 mM NaCl treatment for 1 to 48h. The root radial expression pattern under salt stress conditions for *JAZ5*, *JAZ7* and *JAZ8* genes were obtained from the eFP-Browser web tool to explore gene expression data generated by Geng *et al.* 2013) (http://dinnenylab.info/browser/query). The relative expression levels of epidermis, cortex, stele and endodermis are denoted with color, ranging from low (yellow) to high (red) for 1 to 8 h.

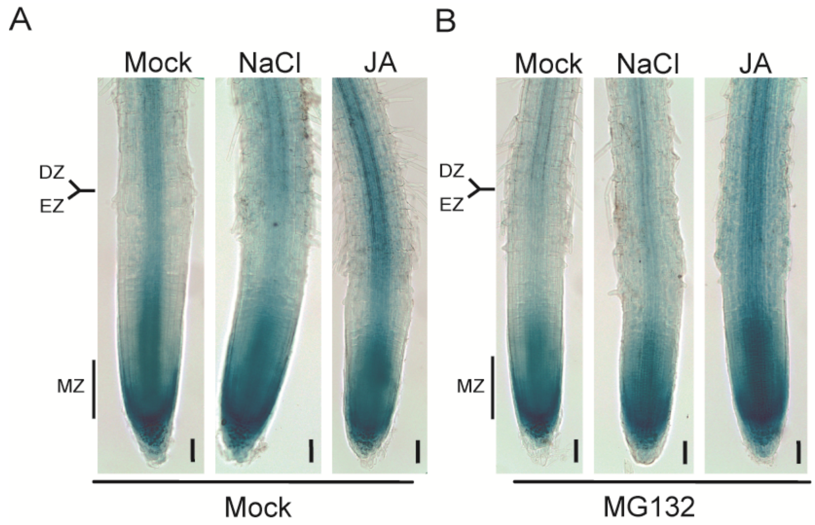

Supplementary Fig. S5. GUS protein levels are not affected by salt stress or by inhibition of proteasome activity in the roots.
(A) 18-d-old *35S::GUS* plants grown in hydroponics were subjected to mock, 150 mM NaCl or 10 nM JA treatments for 6h and stained for GUS activity. (B) Proteasome activity was inhibited before salt stress or JA treatment by preincubating with 50 μM MG132 for 2h. (A) and (B) are representative of three independent experiments with five biological replicates. DZ/EZ denotes boundary region between the differentiation zone (DZ) and the elongation zone (EZ) while MZ the meristematic zone. Scale bar 100 μm.

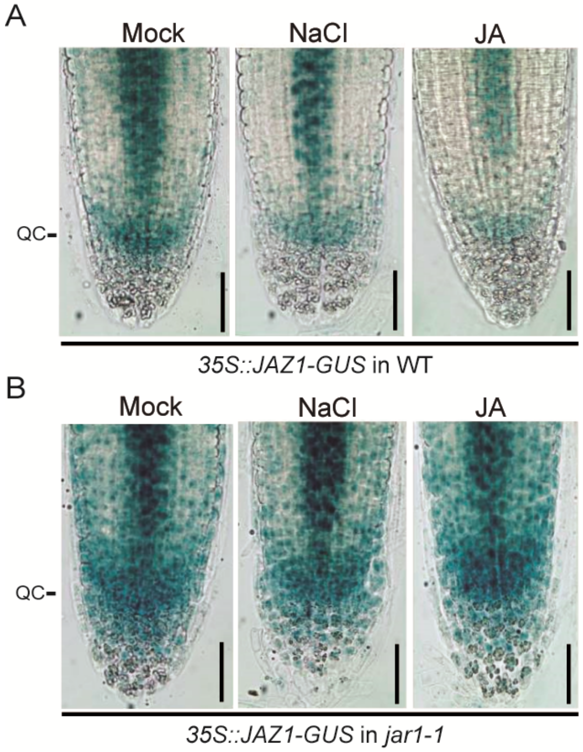

Supplementary Fig. S6. The JA-Ile sensor JAZ1-GUS is destabilized by salt stress in the roots in a JAR1 dependent manner.
(A) 5-d-old seedlings expressing the JA-Ile sensor JAZ1-GUS grown on solid media were subjected to mock, 150 mM NaCl or 10 nM JA treatments for 6h and then stained for GUS activity. (B) Seedlings expressing *JAZ1-GUS* in the *jar1-1* host genetic background. These results are representative of three independent experiments with five biological replicates. QC denotes quiescent center. Scale bar 50 μm.

Supplementary tables

Supplementary Table S1. *JA-related mutants used in this study.*

Mutant	Gene	AGI	Biological process	Reference
aos	*AOS*	At5g42650	JA-biosynthesis	Park *et al.*, 2002
jar1-1	*JAR1*	At2g46370	JA-biosynthesis	Staswick and Tiryaki 2004
coi1-1	*COI1*	At2g39940	JA-signaling	Xie *et al.*, 1998
coi1-2	*COI1*	At2g39940	JA-signaling	Xiao *et al.*, 2004
jai3-1	*JAZ3*	At3g17860	JA-signaling	Chini *et al.*, 2007
	MYC2	At1g32640		
myc2/3/4	*MYC3*	At5g46760	JA-signaling	Fernández-Calvo *et al.*, 2011
	MYC4	At4g17880		

Supplementary Table S2. *AGI locus identifiers with gene description.*

Gene	AGI	Description	Responsiveness
JAZ1	At1g19180	Jasmonate-ZIM-domain protein 1	JA
JAZ2	At1g74950	Jasmonate-ZIM-domain protein 2	JA
JAZ3	At3g17860	Jasmonate-ZIM-domain protein 3	JA
JAZ4	At1g48500	Jasmonate-ZIM-domain protein 4	JA
JAZ5	At1g17380	Jasmonate-ZIM-domain protein 5	JA
JAZ6	At1g72450	Jasmonate-ZIM-domain protein 6	JA
JAZ7	At2g34600	Jasmonate-ZIM-domain protein 7	JA
JAZ8	At1g30135	Jasmonate-ZIM-domain protein 8	JA
JAZ9	At1g70700	Jasmonate-ZIM-domain protein 9	JA
MYC2	At1g32640	Basic-helix-loop-helix (bHLH) family	JA
CML37	At5g42380	Calmodulin-like 37	Salt
PP2C	At3g05640	Protein phosphatase 2C family	Salt
BCB	At5g20230	Blue-copper-binding protein	Salt
TIP41-like	At4g34270	TIP41-like family protein	None, reference gene

Supplementary Table S3. *Nucleotide sequence of the set of primers used in qPCR assays.*

Gene	Sequence	Efficiency (%)
JAZ1	5'-CTTCCTATTGCTAGAAGAGCTTCACTTCACC-3'<br>5'-CCTGTGGTTTGAGGGTTTGAAGACG-3'	93.7
JAZ2	5'-CTTCACTTCATCGGTTCCTTGAGAAGAG-3'<br>5'-CGTGAACTGAGCCAAGCTGGGT-3'	100.9
JAZ3	5'-TGTAATGGCTCCAACAGTGGCATTAC-3'<br>5'-ATTCAGACATTGATCTGCGACAATCTGT-3'	96.4
JAZ4	5'-TAGAGAAACGCAAAGAAAGGGTCATTAAC-3'<br>5'-TTAGTGCAGATGATGAGCTGGAGGAC-3'	102.8
JAZ5	5'-CATCATCGTTATCCTCCCAAGCCA-3'<br>5'-CTTCAATCTTCATAATATCATCTTTATCACCGTCT-3'	93.6
JAZ6	5'-CCCAAGCCAGAGATGGTTGCTCCA-3'<br>5'-GAGTTCAAGGTTTTTGGAAGATTGTCCTTC-3'	104.5
JAZ7	5'-CAATCCCAAACAATTCGACTCGATTT-3'<br>5'-CTATCGGTAACGGTGGTAAGGGGAAG-3'	98.8
JAZ8	5'-CGACCTCATTTCATCATAATCAGCTTCC-3'<br>5'-TTATCGTCGTGAATGGTACGGTGAAGTAG-3'	93.7
JAZ9	5'-TCCTTGGCTCGGTTCTTGGAGAAG-3'<br>5'-GTAGGAGAAGTAGAAGAGTAATTCATTCCACTGG-3'	103.9
MYC2	5'-GTCGGCGTTGATGGATTTGGAGT-3'<br>5'-CTTGCTCTGAGCTGTTCTTGAGTATAGATC-3'	94.9
CML37	5'-AAAGCTGCGTTAGTCTCTTAGG-3'<br>5'-CTATAAACCCGTCTCCGTCAAC-3'	98.3
PP2C	5'-ATGTCAAGAGCATTCGGAGAC-3'<br>5'-CACATCCCATACCCCATCAG-3'	100.5
BCB	5'-CTGGATCAACCCCAAGTACTG-3'<br>5'-GCAGAAACAAAAGCGACCAG-3'	96.4
TIP41-like	5'-GTGAAAACTGTTGGAGAGAAGCAA-3'<br>5'-TCAACTGGATACCCTTTCGCA-3'	97.9

Supplementary Table S4. *qPCR thermal cycle profile used for all genes assayed.*

Stage	Step	Temp (ºC)	Time (min:seg)	Cycles
**Polymerase Activation**	Step 1	95	10:00	1
**PCR Cycling**	Step 1	95	0:10	40
	Step 2	57	0:20	40
	Step 3	72	0:10	40
**Melt Curve**	Step 1	95	0:15	1
	Step 2	65	0:15	1
	Step 3	95	0:15	1

Supplementary Table S5. *Comparison of JAZ genes expression in Arabidopsis roots under salt or JA treatment.*

NaCl				
**Gene**	**AGI**	**Level[1]**	***p*-value**	**Level[2]**
JAZ1	At1g19180	5.2 ± 0.3	0.0001	8.7 ± 1.0
JAZ2	At1g74950	9.6 ± 0.7	0.0003	8.8 ± 1.3
JAZ3	At3g17860	5.2 ± 0.6	0.0070	2.7 ± 0.6
JAZ4	At1g48500	0.5 ± 0.2	0.3327	n.d.
JAZ5	At1g17380	29.2 ± 2.7	0.0004	68.8 ± 3.8
JAZ6	At1g72450	1.8 ± 0.3	0.1850	6.9 ± 1.2
JAZ7	At2g34600	22.6 ± 0.8	< 0.0001	29.1 ± 3.1
JAZ8	At1g30135	30.3 ± 5.7	0.0069	18.1 ± 2.9
JAZ9	At1g70700	9.4 ± 0.7	0.0003	17.3 ± 1.1
**JA**				
**Gene**	**AGI**	**Level[1]**	***p*-value**	**Level[2]**
JAZ1	At1g19180	6.9 ± 0.1	< 0.0001	n.d.
JAZ2	At1g74950	6.0 ± 0.2	< 0.0001	n.d.
JAZ3	At3g17860	5.8 ± 0.7	0.0050	n.d.
JAZ4	At1g48500	1.7 ± 0.3	0.2365	n.d.
JAZ5	At1g17380	24.3 ± 3.9	0.0040	n.d.
JAZ6	At1g72450	4.3 ± 0.5	0.0052	n.d.
JAZ7	At2g34600	11.8 ± 2.3	0.0090	n.d.
JAZ8	At1g30135	13.7 ± 2.3	0.0050	n.d.
JAZ9	At1g70700	8.5 ± 0.6	0.0002	n.d.

[1]The relative transcript levels were quantified by qPCR assays in the roots of WT hydroponically grown 18-d-old plants treated with 150 mM NaCl or 50 μM JA for 3h. Expression in the mock-treated WT roots were arbitrarily set to one. Data is expressed as mean ± SEM of three biological replicates. *p*-values for the up-regulation in the respective treatment are shown. [2]Average transcript levels calculated from root microarray analysis (Affymetrix AHT1 GeneChip) of 18-d-old plants subjected to 150 mM NaCl treatment for 3h (Kilian *et al.*, 2007). The significant differences between non-treated and salt or JA treated plants were determined by a two tailed Student's t-test. n.d. denotes non-determined.

Supplementary Table S6. *Comparison of JAZ genes expression in WT and coi1-2 roots under salt or JA treatment.*

**NaCl**			
**Gene**	**WT**	***coi1-2***	***p-value***
JAZ1	5.2 ± 0.3	2.5 ± 0.0	0.0005
JAZ2	9.6 ± 0.7	1.4 ± 0.1	0.0003
JAZ3	5.2 ± 0.6	2.5 ± 0.0	0.0135
JAZ4	0.5 ± 0.2	0.2 ± 0.0	0.2327
JAZ5	29.2 ± 2.7	8.7 ± 0.9	0.0018
JAZ6	1.8 ± 0.3	1.3 ± 0.1	0.2126
JAZ7	22.6 ± 0.8	3.2 ± 0.3	< 0.0001
JAZ8	30.3 ± 5.7	1.7 ± 0.4	0.0076
JAZ9	9.4 ± 0.7	2.9 ± 0.7	0.0027
Reduction[1]		78.7 ± 0.0	
**JA**			
**Gene**	**WT**	***coi1-2***	***p-value***
JAZ1	6.9 ± 0.1	3.1 ± 0.2	< 0.0001
JAZ2	6.0 ± 0.2	3.4 ± 0.3	0.0020
JAZ3	5.8 ± 0.7	3.0 ± 0.4	0.0214
JAZ4	1.7 ± 0.3	1.4 ± 0.2	0.3442
JAZ5	24.3 ± 3.9	3.8 ± 0.7	0.0068
JAZ6	4.3 ± 0.5	2.5 ± 0.1	0.0182
JAZ7	11.8 ± 2.3	2.4 ± 0.2	0.0145
JAZ8	13.7 ± 2.3	1.9 ± 0.2	0.0065
JAZ9	8.5 ± 0.6	4.0 ± 0.3	0.0024
Reduction[1]		70.6 ± 0.1	

Expression was quantified by qPCR assays in the roots from 18-d-old plants (WT or *coi1-2*) treated with 150 mM NaCl or 50 μM JA for 3h. The transcript levels in the mock-treated WT roots were arbitrarily set to one. Mean ± SEM of three biological replicates are shown. *p*-values for the up-regulation in the respective treatment are shown. [1]Refers to the percentage of reduction for average transcript levels (excluded *JAZ4*) observed in *coi1-2* respect to the wild-type. The significant differences between genotypes under salt or JA treatment were calculated by a two tailed Student's t-test.
